# High-resolution 3D visualization of nanomedicine distribution in tumors

**DOI:** 10.7150/thno.37178

**Published:** 2020-01-01

**Authors:** Jennifer I. Moss, Hervé Barjat, Sally-Ann Emmas, Nicole Strittmatter, Juliana Maynard, Richard J. A. Goodwin, Gert Storm, Twan Lammers, Sanyogitta Puri, Marianne B. Ashford, Simon T. Barry

**Affiliations:** 1 Bioscience, Discovery, Oncology R&D, AstraZeneca, Cambridge, United Kingdom;; 2 Personalised Healthcare and Biomarkers, AstraZeneca, Macclesfield, United Kingdom;; 3 Clinical Pharmacology and Safety Sciences, BioPharmaceuticals R&D, AstraZeneca, Cambridge, United Kingdom;; 4 Department of Pharmaceutics, Utrecht University, Utrecht 3584 CG, The Netherlands; Department of Targeted Therapeutics, University of Twente, Enschede 7500 AE, The Netherlands;; 5 Department of Nanomedicine and Theranostics, Institute for Experimental Molecular Imaging, RWTH Aachen University, Aachen 52074, Germany; Department of Pharmaceutics, Utrecht University, Utrecht 3584 CG, The Netherlands; Department of Targeted Therapeutics, University of Twente, Enschede 7500 AE, The Netherlands;; 6 Pharmaceutical Sciences, BioPharmaceuticals R&D, AstraZeneca, Cambridge, United Kingdom;; 7 Pharmaceutical Sciences, BioPharmaceuticals R&D, AstraZeneca, Macclesfield, United Kingdom.

**Keywords:** EPR, nanomedicine, distribution, µCT imaging, tumor microenvironment, vasculature.

## Abstract

To improve the clinical translation of anti-cancer nanomedicines, it is necessary to begin building specific insights into the broad concept of the Enhanced Permeability and Retention (EPR) effect, using detailed investigations of the accumulation, distribution and retention of nanomedicines in solid tumors. Nanomedicine accumulation in preclinical tumors has been extensively studied; however, treatment efficacy will be heavily influenced by both the quantity of drug-loaded nanomedicines reaching the tumor as well as their spatial distribution throughout the tumor. It remains a challenge to image the heterogeneity of nanomedicine distribution in 3 dimensions within solid tumors with a high degree of spatial resolution using standard imaging approaches.

**Methods:** To achieve this, an ex vivo micro computed tomography (µCT) imaging approach was developed to visualize the intratumoral distribution of contrast agent-loaded PEGylated liposomes. Using this semi-quantitative method, whole 3-dimensional (3D) tumor liposome distribution was determined with 17 µm resolution in a phenotypically diverse panel of four preclinical xenograft and patient-derived explant (PDX) tumor models.

**Results:** High-resolution ex vivo μCT imaging revealed striking differences in liposome distribution within tumors in four models with different vascular patterns and densities, stromal contents, and microenvironment morphologies. Following intravenous dosing, the model with the highest density of pericyte-supported vessels showed the greatest liposome accumulation, while the model with vessels present in regions of high α-smooth muscle actin (αSMA) content presented with a large proportion of the liposomes at depths beyond the tumor periphery. The two models with an unsupported vascular network demonstrated a more restricted pattern of liposome distribution.

**Conclusion:** Taken together, vessel distribution and support (the latter indicative of functionality) appear to be key factors determining the accumulation and distribution pattern of liposomes in tumors. Our findings demonstrate that high-resolution 3D visualization of nanomedicine distribution is a useful tool for preclinical nanomedicine research, providing valuable insights into the influence of the tumor vasculature and microenvironment on nanomedicine localization.

## Introduction

The majority of oncology drugs delivered using a nanomedicine carrier have been developed primarily to alter the drug's pharmacokinetic profile and biodistribution to improve its therapeutic index. Despite significant investment in nanomedicine development over the last two decades, successful clinical translation of these nanomedicines has been disappointing 1-7. Novel nanomedicines under development and the current generation of nanomedicines on the market have been assessed using established, basic preclinical approaches. These include characterization of physicochemical properties, *in vitro* cell-based assays, and a limited number of *in vivo* pharmacokinetic/biodistribution and efficacy studies in xenograft tumor models 1, 2, 5.

Development of nanomedicines is often based on the premise that there is potential to accumulate and achieve prolonged retention in solid tumors via the Enhanced Permeability and Retention (EPR) effect. It is typically assumed that the EPR effect is a universal property of solid tumors and key to nanomedicine anti-cancer agent efficacy. However, more recently this assumption is being challenged 1. Changes in systemic plasma profiles and therapeutic index are also being recognised as potential critical drivers of nanomedicine efficacy and clinical success 8, and it has been shown that delivery system size and shape can alter carrier plasma kinetics and tumor accumulation 9, 10. Solely relying on the proposed EPR effect to deliver enhanced efficacy in tumors is still debatable and challenged by experts, as evident from various clinical trial readouts showing minimum benefit in efficacy 1.

Nanomedicine accumulation in tumors has been demonstrated, but has been shown to be highly heterogeneous both clinically and preclinically, with variability between different tumors (even within a single patient) and also within an individual tumor 1, 6, 7, 11-14. While variation in tumor features may not alter the peripheral pharmacokinetics of nanocarriers, the tumor microenvironment significantly influences their intratumoral accumulation, distribution and retention. The pattern of nanomedicine and drug localization/disposition throughout the whole 3-dimensional (3D) tumor mass - henceforth referred to as distribution - will impact local drug concentrations and the levels of target engagement. Non-uniform accumulation and distribution may lead to heterogeneous efficacy across discrete areas of the tumor, impacting the overall therapeutic outcome. Consequently, to design more effective anti-cancer nanomedicinal therapeutics, it is necessary to build insight into how certain tumor features influence delivery system accumulation, distribution and retention.

As increasing numbers of nanomedicines, with varying physicochemical attributes, progress towards clinical development, it is critical to understand how these systems (agnostic of drug) accumulate in and distribute within tumors, and identify the key factors influences these processes 1, 15. Examining nanomedicine distribution within tumors is important for two reasons. Firstly, understanding how a specific delivery system accumulates and distributes in diverse tumor microenvironments is important for disease or patient selection and may influence the choice of delivery system for a therapeutic payload. Patients with specific microenvironment features may be more (or less) likely to receive therapeutic benefit from a nanomedicine. Enriching treatment groups for patients with tumors likely to be amenable to nanomedicinal therapeutics is important for clinical success, particularly in early stage clinical development. Secondly, disease-focused design of nanomedicines may be a more translatable approach to development than standard approaches that focus on development of the delivery system agnostic of its intended patient population. A disease-focused approach optimises the physicochemical properties, such as size and drug release rate, of novel carrier systems based on the dominant features of the tumor microenvironment of that disease 1.

Standard preclinical nanomedicine research uses a composite of histology, whole tissue bioanalysis, and 2-dimensional (2D) imaging to gain confidence that the nanomedicine has accessed the tumor (i.e., accumulation) and achieves a prolonged duration of drug exposure (i.e., retention). These techniques have been useful to identify that nanomedicine accumulation within preclinical and clinical tumors is highly heterogeneous. With methods such as whole tissue bioanalysis or standard luminescent imaging, no spatial distribution or heterogeneity data are obtained. Moreover, the typical approaches to evaluate the accumulation of nanomedicines are not ideal when comparing multiple delivery systems that have been developed to function with different imaging modalities 16. When assessing spatial distribution, switching between imaging platforms can make it challenging to drive robust comparisons between datasets. Finally, techniques that require whole tumors to be analyzed as composites of tens or hundreds of 2D sections can become labor-intensive and low throughput.

The ideal workflow would capture the heterogeneity in 3D distribution across the whole tumor with sufficient resolution to correlate distribution patterns with classical histology and other 2D techniques. 2D fluorescent microscopy is a common technique used to assess delivery system distribution using fluorophore-labelled drug carriers, or systems delivering naturally fluorescent drugs, such as doxorubicin. Recent advances in 2D spatial analytical techniques, such as mass spectrometry imaging (MSI), have significantly enhanced our ability to study delivery system and drug localization. This mapping technique achieves micron-level resolution, including the simultaneous detection of multiple analytes. However, even MSI is limited by slow data acquisition, and requires compilation rendering of multiple 2D slices to gain insight into 3D architecture and localization. Hence, performing detailed 3D assessment of nanomedicine distribution is challenging, particularly when needing to acquire a sufficient number of data points to analyse distribution over time in multiple models and across platforms.

The 3D distribution of nanomedicines and tumor vasculature can be assessed by µCT imaging 11, 13, 17-19. Previous investigations have addressed specific questions using imaging at either higher resolution (1 µm), but with lower model diversity 11, or lower resolution (100 µm) using live imaging in two different tumor models 20. However, to support nanomedicine evaluation and screening, a balance of throughput (i.e., sample number) and resolution (i.e., level of detail) to enable correlation with histology is required. To overcome these challenges, we present here an ex vivo µCT imaging method to enable cost-effective assessment of liposome distribution in a range of tumor phenotypes. By performing ex vivo imaging of freshly excised tissue, it is possible to decrease the cost and complexity of the technique, and also to avoid subjecting live animals to prolonged imaging procedures under anesthesia, as is necessary for dynamic contrast-enhanced magnetic resonance imaging (DCE-MRI). Moreover, high-resolution ex vivo 3D µCT imaging of whole tumors is non-destructive and can be coupled to subsequent complementary analyses. The method presented in this paper achieves a resolution of 17 µm at a 1 h scan time per tumor. Other lower throughput and/or higher resolution analyses are limited by the number of tumors that can be assessed. With these methods it is not possible to image a sufficient number of samples to encompass a diverse panel of models, thereby preventing a robust examination of the link between tumor features and delivery system distribution. Moreover, in contrast to the method described here, analyses with limits on sample numbers are also not amenable to the evaluation of multiple systems in a single model over time.

We here set out to evaluate whether ex vivo µCT imaging can provide high-resolution whole-tumor 3D visualization of nanomedicine distribution, with sufficient detection sensitivity to identify variation in liposome distribution in tumors and semi-quantitatively assess relative liposome accumulation over time. We examined a panel of PDX and xenograft tumor models with sufficient phenotypic diversity to build new insights into correlations between specific tumor features and liposome distribution. These data will help guide the choice of delivery system for specific patient populations with defined tumor microenvironments. For example, knowing which type of platform achieves homogeneous distribution in highly cellular, low stroma tumors with a moderate vessel density makes it possible to match the right therapy to patients with specific disease features, which should enhance clinical effect.

## Methods

### Tumor models

All animal studies were conducted in accordance with UK Home Office legislation, the Animal Scientific Procedures Act 1986, and the AstraZeneca Global Bioethics policy. All experimental work is outlined in project licence 40/3483, which has gone through the AstraZeneca Ethical Review Process. All mice weighed more than 18 g at the time of the first procedure and were purchased from Charles River UK (SCID mice) or Harlan UK (nude mice). Human cancer cell lines were implanted subcutaneously onto the left flank of mice as follows: female nude mice were implanted with Calu-6 cells (1 x 10^6^ cells/mouse in 50% Matrigel) or H358 cells (5 x 10^6^ cells/mouse in 50% Matrigel); female SCID mice were implanted with Calu-3 cells (8.8 x 10^6^ cells/mouse in 30% Matrigel); E77 or E35CR tumors (3 x 3 mm fragments of freshly excised tumor) were passaged into castrated male SCID mice. Tumor growth was monitored once to twice weekly using intersecting calliper measurements; tumor volume was calculated from 3.14 x length x width^2^/6000, where length is the longer dimension and width is the shorter dimension. Body weights were recorded at the time of tumor measurement. When tumor volumes reached ~500 mm^3^, mice were randomised based on tumor volume to ensure an equal volume distribution across groups. Clinical condition and body weights were monitored daily during the dosing period.

Nanotrast-CM liposomes were purchased from NanoVista (Toronto, Canada). Briefly, liposomes (hydrodynamic diameter of 90-100 nm) were composed of DPPC, cholesterol and DSPE-PEG_2000_ (55:40:5 mole percent ratio) and encapsulated iohexol-based contrast agent Omnipaque™ (Nycomed Imaging, Oslo, Norway) with a final iodine concentration of 55-56 mg/mL 17, 21, 22. Liposomes were administered as supplied, and also contained a magnetic resonance imaging agent that is not of relevance to these studies. Mice were dosed intravenously (IV) via the lateral tail vein at 0.1 mL liposome solution/10 g mouse bodyweight or intratumorally (ITu) with 25 µL of liposome solution.

### Immunohistochemistry (IHC)

Immunohistochemical staining for CD31 and alpha smooth muscle actin (αSMA) was completed to assess the vessel distribution and stromal content, respectively, in tumors. Formalin-fixed paraffin-embedded tumor sections (4 µm) were dewaxed with xylene and rehydrated through graded ethanols to water. Antigen retrieval was performed in pH 6 citrate buffer at 110°C for 5 min in a RHS histoprocessor microwave. Endogenous enzyme activity was blocked with 3% hydrogen peroxide. For αSMA staining: slides were incubated with serum free protein block (Dako) for 20 min before the primary antibody (anti-actin α-smooth muscle (clone 1A4); 1:1000; Sigma) was added for 30 min at room temperature. The Dako Envision+ (anti-mouse) peroxidase-labelled polymer system was applied for 30 min. For CD31 staining: slides were incubated with Avidin Block then Biotin Block (10 min each; Vector Elite ABC Kit (Rabbit)). 20% normal goat serum was applied for 20 min before the primary antibody (CD31 (CHG-CD31-P1); 1:1000; AstraZeneca custom antibody generation) was added for 1 h at room temperature. Secondary antibody (biotinylated anti-rabbit IgG; 1:200) was applied for 30 min, before the tertiary antibody (Vector Elite ABC) was added for 30 min. For both αSMA and CD31, the slides were then incubated with DAB for 10 min. Slides were counterstained with Carazzi's hematoxylin, dehydrated through graded ethanols to xylene, and coverslipped. Snapshots of scanned tumor sections were taken using ImageScope software (v11.2.0.780; Aperio).

### CT scanning and image analysis

#### CT acquisition

At the desired time point, the animal was euthanized via cervical dislocation with secondary confirmation of death and the tumor was excised and placed into a small plastic tube which was attached to a plastic holder. The tumor was then placed in the center of the scanner and then scanned immediately at room temperature. All scans were acquired on the CT module of an Inveon PET/CT preclinical imaging scanner (Siemens, Knoxsville, USA). Data were acquired using Inveon Acquisition Workplace (IAW) software (Siemens version 1.5). The acquisition parameters were: X-ray tube voltage 80 KeV and current 500 µA, 720 projections of 2 s exposure each were acquired over 360 degrees, the total acquisition time was 43 minutes. The X-ray detector pitch was calculated to be 16.6144 µM using a projection bin factor of 1 and a settling time between projections of 500 ms.

#### CT reconstruction and analysis

The X-ray µCT data was reconstructed using Inveon Reconstruction software (Siemens) version 2.2.0 using the filtered back projection method. The resulting 3D image data sets had a nominal isotropic resolution of 16.6144 µM. The reconstructed 3D image data sets were processed by Image J (NIH, Bethesda, USA; http://imagej.nih.gov.ij/) using in-house macros to set non-tumor voxels to background. Tumor depth maps were computed using the “Local Thickness” ImageJ plugin (Optinva, Inc, Seattle, USA). Maximum intensity projection images, where “each of whose pixels contains the maximum value over all images in the stack at the particular pixel location” were generated using Image J (NIH). Image processing was done using R2.

To estimate the amount of contrast agent present in the tumor, solutions containing the iodinated liposomes in different concentrations ranging from a minimum of 0.55 mg/mL to a maximum of 27.5 mg/mL were prepared. These samples were scanned and the image reconstructed using the same method as that for the excised tumors. The reconstructed 3D X-ray µCT data sets were used to measure the mean attenuation in a large 3D region of interest for each sample and a linear fit was performed to yield the attenuation rate. The change in X-ray attenuation was divided by the change in iodine concentration.

To estimate the volume of distribution of contrast agent within the tumor we used the histogram of the X-ray µCT attenuation values that we measured in the tumor. It is assumed that because the contrast agent is encapsulated in a liposome it does not distribute homogeneously through the tumor and it is assumed that large parts of the tumor do not contain any contrast agent; therefore, the lower portion of the attenuation histogram represents parts of the tumors without liposomes and it can be used to estimate the mean and standard deviation of the distribution of the attenuation values for those parts. In practice, the part of the histogram containing the lower attenuation values is discarded as it corresponds to the voxels affected by the partial volume effects or small amounts of tumor motion during the duration of the scan or those incorrectly assigned as tumor. Therefore, the part used for the estimation of the mean and standard deviation via filling to a Gaussian function is from the 5^th^ to 95^th^ percentile (median) when injections were performed IV and from the 10^th^ to 50^th^ percentile (median) when injections were performed ITu. The resulting fitted curve is subtracted from the experimental histogram counts and the resulting differential histogram of voxel counts is integrated over a relevant range of attenuation values and converted into a volume of voxels deviating from the Gaussian distribution. In all cases the upper limit of the range of attenuation is the maximum attenuation measured in the tumor. The lower limit is set differently depending on the route of injection (IV or ITu); for samples that underwent IV injection the lower limit is set to the attenuation value greater than or equal to that of the 99^th^ percentile for which the differential histogram is greater than zero. For ITu injection it is set to the estimated mean attenuation.

To estimate the injected dose within the tumor the mean X-ray attenuation values of the Gaussian fit curve described earlier is set as the attenuation value at which we assume there is no iodine present. Any non-zero voxel counts in the differential histogram can be attributed to the presence of iodinated liposomes, the concentration of which can be estimated by using the difference in attenuation between the estimated mean attenuation value and the attenuation values for those voxels divided by the attenuation rate. The voxel counts in the differential histogram obtained during the estimation of the volume of distribution, together with the attenuation difference from the estimated mean attenuation values combined with the scalar values representing change in attenuation per change in iodine concentration are used to estimate the iodine content for each differential bin. The estimated total iodine content is obtained by summing the entire content over the relevant range of bins in the differential histogram. In all cases the upper limit of the range is the maximum attenuation measured in the tumor. The lower limit as before depends on the route of injection (IV or ITu). For samples that underwent IV injection it is set as the first attenuation value greater than or equal to that of the 99^th^ percentile for which the differential histogram is greater than zero. For ITu injection it is set as the estimated mean attenuation.

For surface rendering of the regions of high attenuation, the voxels from the segmented tumor image were classified into three groups: non-tumor voxels, high attenuation voxels and all other voxels. The surface of the tumor was rendered in semi-transparent grey and that of the regions of high X-ray attenuation in solid yellow using the R package “rgl” (https://r-forge.r-project.prg/projects/rgl/). In addition, the regions of high attenuation were encoded as a function of tumor depth in 1 mm shells. The result was then rendered with the surface of the tumor in semi-transparent grey and the high attenuation regions displayed with a depth-dependent color.

### Detection of iohexol in tumor sections via desorption electrospray ionisation-mass spectrometry imaging (DESI-MSI)

After µCT imaging, tumors were immediately snap-frozen. These tissues were cryo-sectioned using a Leica CM3050 S cryostat (Leica Microsystems) to a thickness of 14 µm, thaw mounted onto Superfrost microscope slides and stored at -80°C until analysis. DESI-MS imaging experiments were performed on a Thermo Scientific Q-Exactive instrument (Bremen, Germany) equipped with a 2D automated DESI source from Prosolia Inc. (Indianapolis, USA) using a home-built sprayer assembly as described previously 23. Imaging of iohexol and DSPE-PEG liposome constituents were performed in positive ion mode in full scan using a mass range of m/z 750-1000 and a spatial resolution of 130 µm. Iohexol was detected as [M+K]^+^ at m/z 859.8274 and liposomes were detected as multiply charged PEG polymer (m/z 946.1929, z = 3 used for visualization). Analysis was performed at R = 70,000 mass resolving power (5E6 AGC target, prescan on) and 50 V S-Lens Voltage. Methanol/water (95: 5 v/v) was used as electrospray solvent at 4.5 kV spray voltage and a flow rate of 1.5 µL/min. Solvent was delivered using a Dionex Ultimate 3000 stand-alone nanoLC pump (Thermo Scientific). Nitrogen N4.8 was used as nebulising gas at a pressure of 7 bar. Distance between sprayer to sample surface was 1.5 mm while distance between sprayer and MS inlet was 7 mm. The spray angle was set to 75° while collection angle was set at 10°. The capillary temperature was set to 320°C. Data were converted into centroid .imzML format using imzML converter version 1.1.4.5 24 and visualized using MSiReader v0.05 25 using ± 0.005 Da as bin size. All intensities shown are raw ion intensities. 1^st^ order linear interpolation was used for image generation. Samples were stained with hematoxylin and eosin post-DESI analysis and digitalised using a Leica Aperio AT2 slide scanner (Leica Microsystems).

## Results

### Selection of nanomedicine

To image nanomedicine distribution in 3D, PEGylated liposomes were selected as an archetypal drug delivery system of ~100 nm diameter which has been well-characterised *in vitro* and *in vivo*. Commercially available PEGylated liposomes containing an iodine-based CT contrast agent (iohexol) were used 17, 21, 22. The DPPC:cholesterol:DSPE-PEG_2000_ liposomes show a prolonged circulation time *in vivo* as the PEG coating reduces the rate of protein opsonisation and thereby slows and reduces plasma clearance. As a result, the encapsulated iohexol has a nearly 100-fold longer half-life relative to free iohexol 22. The formulation was stable, with minimal release of the iodine-based contrast agent iohexol, *in vitro* and *in vivo* over the duration of the intended study time course. Systemic enhancement of iohexol detection was demonstrated for up to 3 days post-dose *in vivo* 22. Free iohexol is rapidly cleared *in vivo*, with a half-life of 12.3 min 22. As a result, any released iohexol would provide minimal contribution to any µCT enhancement detected, and the Nanotrast-C/M liposomes are well-suited to the development of high-resolution imaging methodology as a measure of liposome distribution.

### Optimization of scanning parameters

The optimal scanning parameters to detect iohexol in animal tissues were determined. To establish a limit of detection, saline or free iohexol were administered IV or ITu (at an equivalent iodine dose as delivered with iohexol-loaded Nanotrast-C/M liposomes) and did not show deviation from background attenuation in tumors at 30 min post-injection (Supplementary Fig. 2). This confirmed that the imaging and analysis would only detect encapsulated iohexol. Should burst release of the entire dose of encapsulated iohexol occur, it would fall below the limit of detection before 30 min post-dose.

### Validation of ex vivo µCT imaging with mass spectrometry imaging

**Figure 1:**
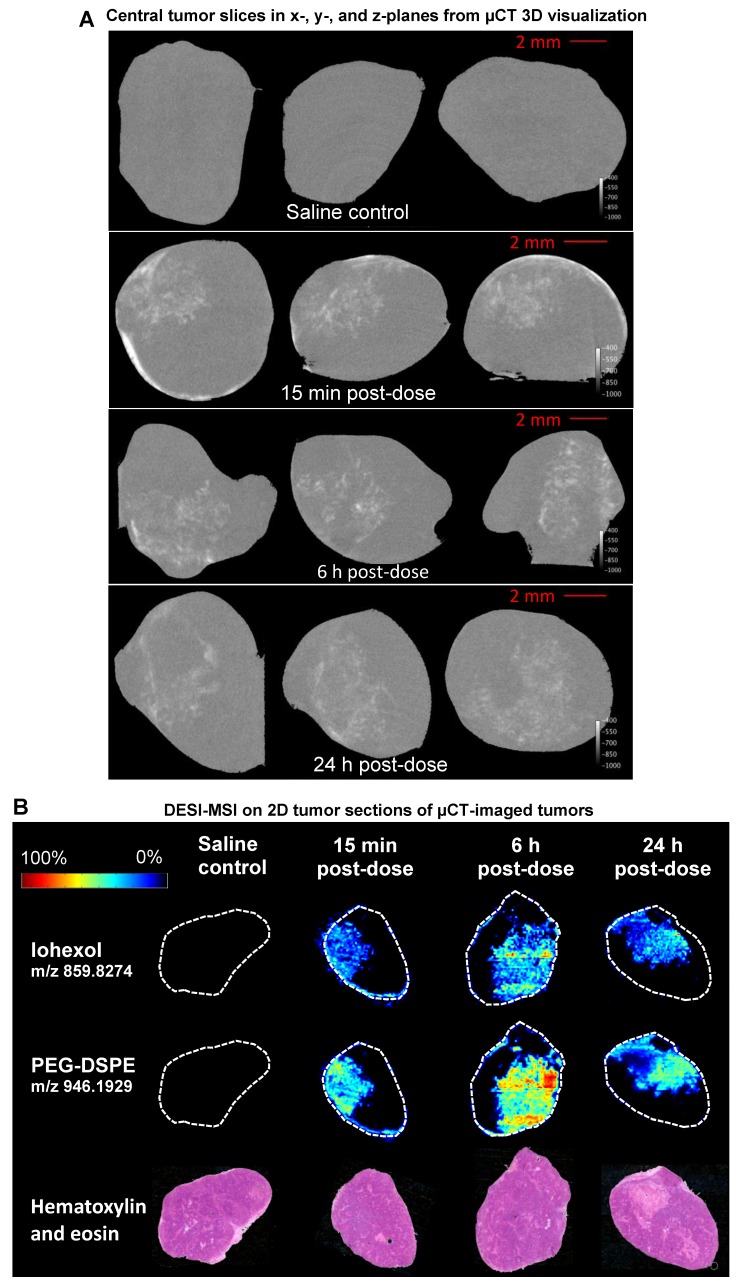
Use of the well-established 2D spatial detection technique DESI-MSI to validate the ex vivo µCT imaging for 3D visualization of nanomedicine distribution in tumors. Tumors were collected at 15 min, 6 h or 24 h post-ITu dose and imaged via ex vivo µCT to generate 3D information. Tumors were then immediately snap-frozen and subsequently sectioned and analyzed via DESI-MSI to assess the correlation of iohexol and liposome distribution in 2D sections of the imaged tumors. A) Distribution of iohexol in x-, y- and z-plane images (left to right, respectively) extracted from the center of the 3D µCT visualization. B) Distribution of iohexol and liposome constituent DSPE-PEG in the imaged tumors, as detected using DESI-MSI on 2D tissue sections from the center of the tumor. Hematoxylin and eosin stain of each tumor also shown.

DESI-MSI analysis of non-small cell lung adenocarcinoma xenograft model (H358) tumors was used to validate the high-resolution µCT nanomedicine visualization approach. Following ITu dosing, tumors were imaged ex vivo using µCT, which is non-destructive, maintains tissue integrity, and permits additional processing for further analyses. After µCT imaging, tumors were halved and snap-frozen. Subsequently, central slices of the frozen tumors were prepared for DESI-MSI to map the distribution pattern of the iohexol in the tumor. MSI is a sensitive, specific, well-established 2D imaging method utilised for high-resolution spatial mapping. The unique distribution patterns achieved with ITu dosing allowed us to assess whether a general concordance was observed in the iohexol distribution pattern detected using both techniques in each of the matched tumors. By visual assessment, we detected the same distribution pattern of iohexol between the two methods for matched tumors at 3 different time points. Figure 1A depicts single 'slices' of the 3D tumor image generated from ex vivo µCT imaging while the matched DESI-MSI analysis on 2D tissue sections from the tumor center are shown in Figure 1B. The 2D slices of µCT-imaged tumors show one x-, y- and z-plane from the center of the tumor image (Fig. 1A). Both µCT and MSI showed that a 25 µL dose of liposomes administered intratumorally results in liposome presence in the tumor, with roughly one-third of the tumor being exposed to high concentrations of liposomes.

Moreover, DESI-MSI detection showed a very strong correlation between the distribution of iohexol and the liposome constituent DSPE-PEG (Fig. 1B). Together these findings suggest that this novel ex vivo µCT imaging and analysis method is a useful tool for visualizing liposome distribution in 3D. Following ITu administration of liposomes to H358 tumors, the liposome distribution patterns detected using µCT and MSI were consistent between the methods in 3 different tumors collected at 15 min, 6 h or 24 h time points post-dose. A video showing a rotating view of a 3D visualization of liposomes in a tumor following ITu dosing is presented in Supplementary Figure 3.

### Tumor microenvironment features across panel of preclinical models

**Figure 2:**
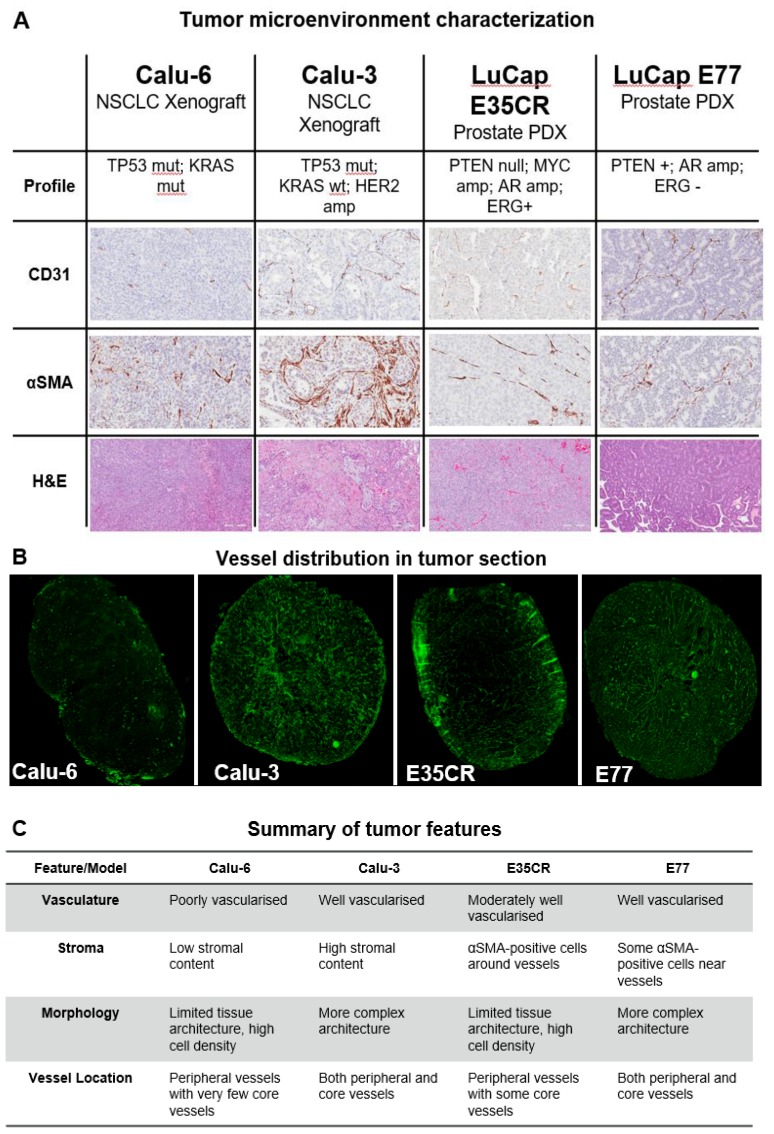
A) Diversity in tumor microenvironment features (vasculature (CD31), stroma (αSMA), and morphology (hematoxylin and eosin)) across four preclinical tumor models. A representative 20x snapshot is shown. B) Representative images from four models showing tumoral vessel location. C) Summary of tumor microenvironment features across models.

The structure of the tumor microenvironment can influence nanomedicine efficacy in preclinical models 26. Nanomedicine distribution was compared in four tumor models with diverse microenvironments. Immunohistochemical staining (Fig. 2A) revealed variation in vascular (CD31) and stromal (αSMA) density between models, as well as in morphology (hematoxylin and eosin staining) and blood vessel distribution (Fig. 2B). Two human non-small cell lung carcinoma (NSCLC) cell-line derived xenograft models (Calu-3 and Calu-6) and two *in vivo* serially passaged human prostate patient-derived xenograft (PDX) tumor models (E35CR and E77) were examined. The distribution of functional vessels within the tumor is thought to be one of the primary features influencing nanomedicine localization. The distribution of CD31-positive (vascular) cells, and stromal cells, across a whole tumor section is shown for all models (Supplementary Fig. 1). The Calu-3 cell line-derived xenograft model is highly vascularized and has a high stromal content, with vessels located throughout both the tumor core and periphery. In contrast, the cell line-derived Calu-6 tumors present with low stromal and vascular density. Their vasculature is primarily located in the periphery of the tumor, with a very low prevalence of blood vessels in the tumor core. The E35CR PDX tumors are well-vascularized, with pericyte-covered vessels, but an absence of other αSMA-positive cells. The vessels are most abundant in the periphery, and less dense in the tumor core. The E77 PDX tumors are also serially passaged *in vivo* and are highly vascularized, but, in contrast, present with vessels throughout the tumor. They show some αSMA-positive cells near vessels but also lack a significant stromal content. The key microenvironment features of each model are summarized in Fig. 2C.

### Visualization of liposome distribution in tumors using ex vivo µCT imaging

**Figure 3:**
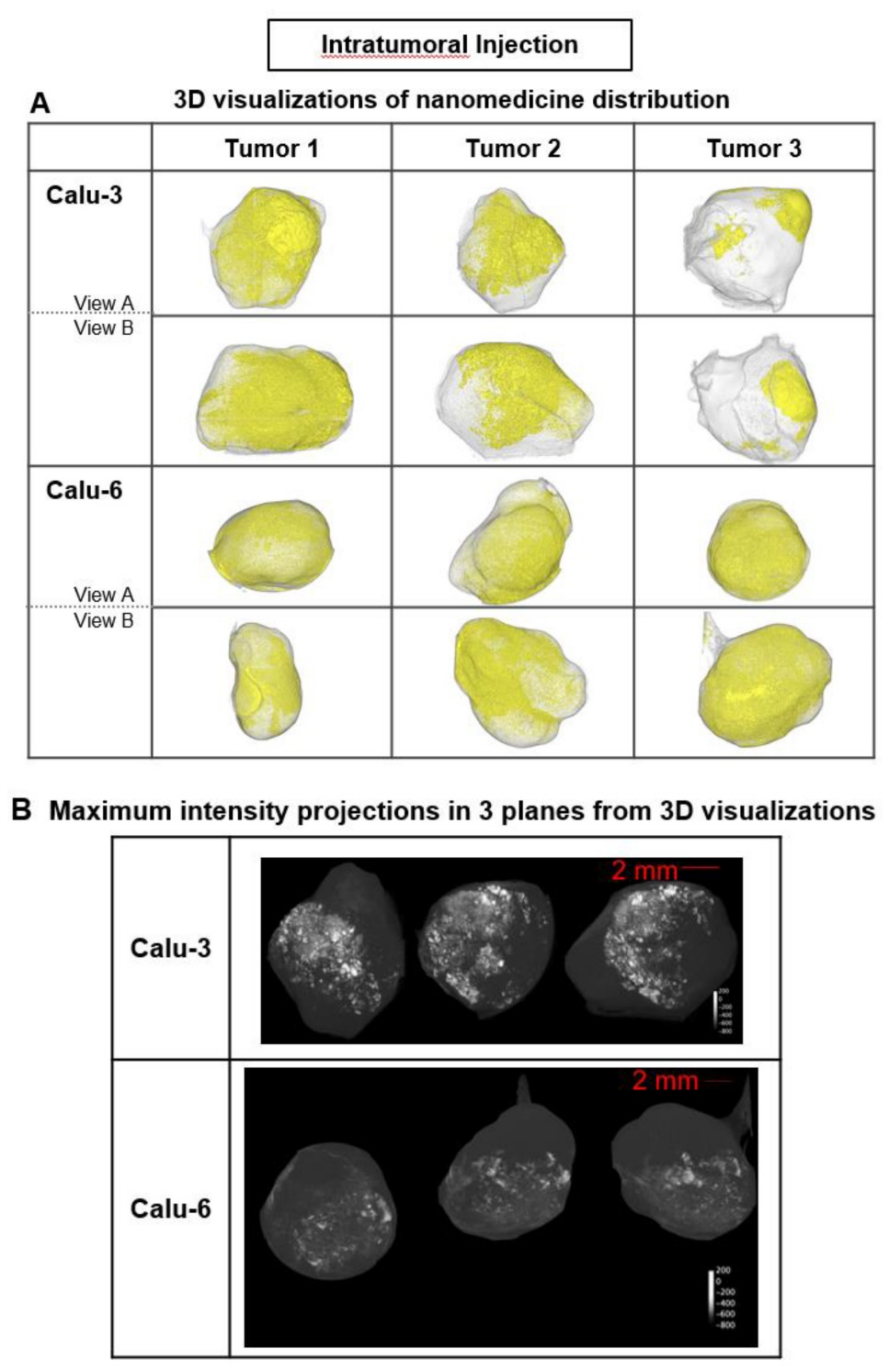
Liposome accumulation and distribution in preclinical tumor models following intratumoral injection. A) Liposome distribution in 3D at 6 h post-ITu dose in Calu-3 and Calu-6 tumors. Two views of each tumor shown. B) Maximum intensity projections of liposome distribution in 1 dimension at 6 h post-ITu dose for the x- (left), y- (middle), and z-plane (right) in representative Calu-3 (left) and Calu-6 (right) tumors.

**Figure 4:**
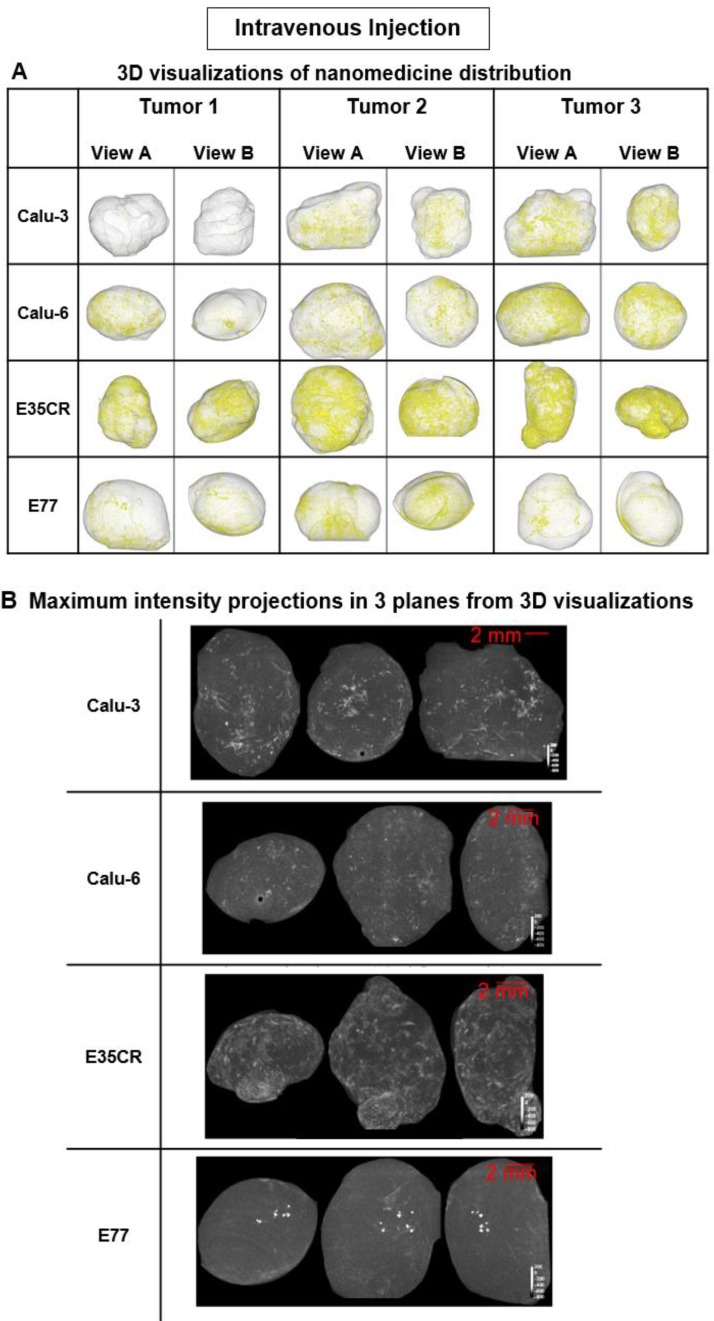
Liposome accumulation and distribution in preclinical tumor models following intravenous injection. A) Liposome distribution in 3D at 24 h post-IV dose in Calu-3, Calu-6, E35CR and E77 tumors. B) Maximum intensity projections of liposome distribution in 1 dimension at 24 h post-IV dose for the x- (left), y- (middle), and z-plane (right) in representative Calu-3, Calu-6, E35CR and E77 tumors.

In each tumor model, 3D high-resolution μCT visualization of liposomes administered via intratumoral (ITu; Fig. 3) or intravenous (IV; Fig. 4) injection was performed. First, the distribution and semi-quantification of liposomes dosed ITu in Calu-3 and Calu-6 tumors was assessed to confirm the analysis parameters. High levels of contrast agent were present and retained in the tumors. After a single ITu dose, the Calu-3 and Calu-6 tumors showed similar patterns of distribution (Fig. 3B), despite their different stromal and vascular morphologies. The liposomes were detected in higher relative concentrations in the Calu-3 tumors than Calu-6 tumors. ITu administrations resulted in focalized areas of highly concentrated liposomes, alongside large regions of the tumor devoid of liposomes. As might be expected, the liposome-rich areas were typically near the injection site and around the tumor periphery. These regions covered approximately one-third of the tumor in 3D in both models.

Following IV administration, substantially lower quantities of liposomes were detected in tumors relative to ITu dosing, as expected. Nonetheless, the visualization method proved to be sufficiently sensitive to show that the pattern of liposome distribution after IV dosing was significantly different across the four tumor models. At 24 h post-dose, the most homogeneous distribution was observed in the E35CR model, while concentrated, highly focalized liposome distribution was seen in the E77 tumors (Fig. 4B). The vessels in the E35CR tumors are associated with a pericyte layer, suggesting they may be more mature and perhaps more functional than typical immature angiogenic vessels, and could provide better blood flow and nanomedicine delivery than in other tumor types.

### Visualization of liposome distribution time course

**Figure 5:**
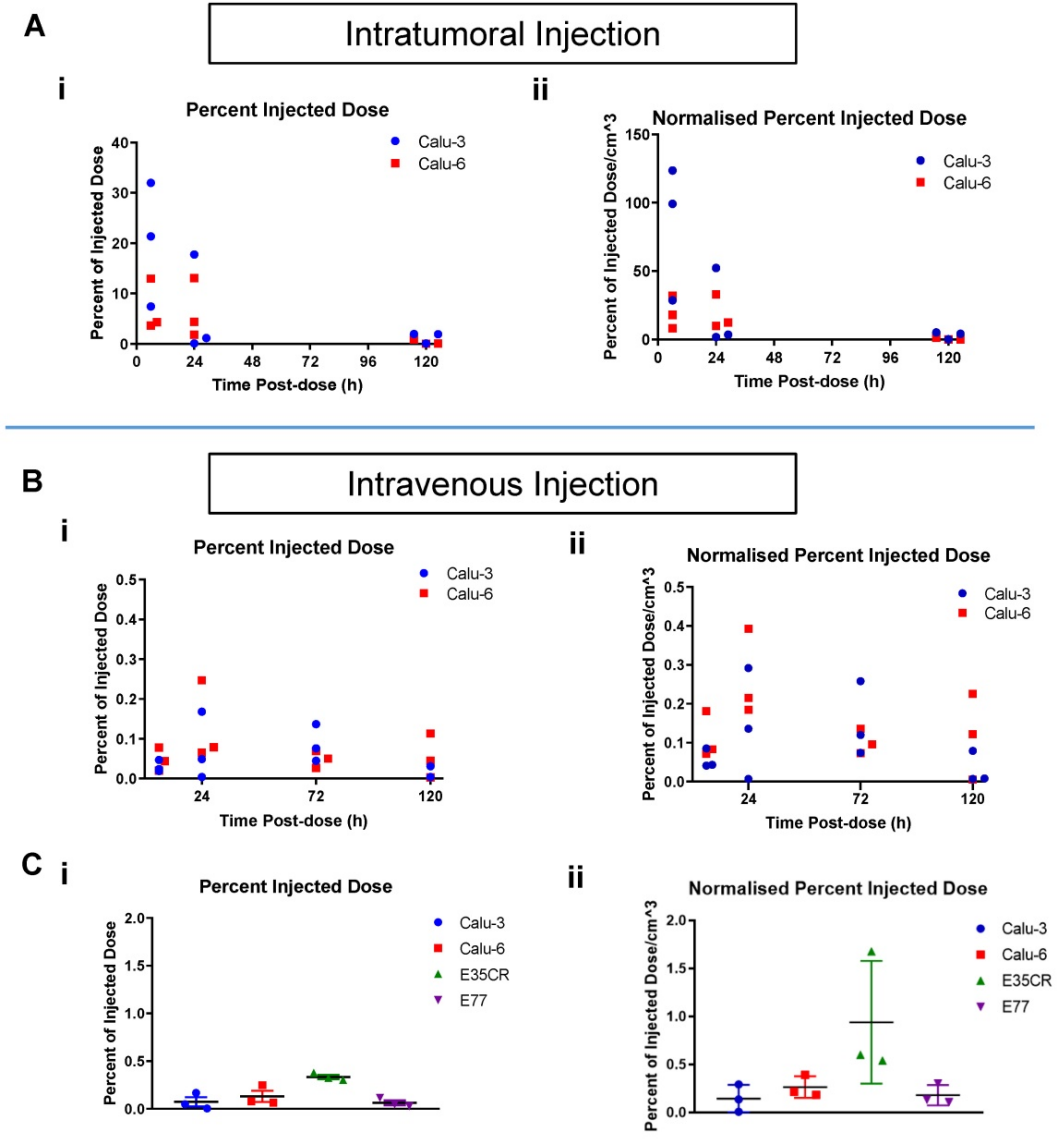
A) Time course of percent of injected liposome dose per tumor (i) and per volume (cm^3^) (ii) in Calu-3 and Calu-6 tumors, collected and imaged at 6 h, 24 h, and 120 h post-ITu dose. B) Time course of percent of injected liposome dose per tumor (i) and per volume (cm^3^) (ii) in Calu-3 and Calu-6 tumors, collected and imaged at 6 h, 24 h, 72 h, and 120 h post-IV dose. C) Percent of injected liposome dose per tumor (i) and per volume (cm^3^) (ii) at 24 h post-IV dose in the panel of tumor models.

To understand if liposome accumulation varied over time after ITu dosing in the Calu-3 and Calu-6 models, individual tumors were imaged at time points between 6 h and 120 h post-dose (Fig. 5). After ITu liposome administration (Fig. 5A), the peak liposome accumulation was observed at the earliest time point (6 h) post-dose (Fig. 5A i). The accumulation at the 6 h time point was significantly higher in the Calu-3 tumors than in the Calu-6 tumors. In the highly stromal Calu-3 tumors, the liposome dose present in the tumors at 24 h was substantially lower than at 6 h. This may suggest that the complex stromal network in Calu-3 tumors contributes to the maintenance of tumor structure and permits greater nanomedicine movement compared with other tumor models (Fig. 2). A less significant decline in liposome content from 6 h to 24 h was observed with the poorly vascularized Calu-6 tumors, though the dose present at 6 h was also lower in this model. In both models, very little or no contrast agent could be detected at 120 h post-dose.

When a time course assessment was completed in the Calu-3 and Calu-6 tumors following IV liposome administration (Fig. 5B), tumor accumulation peaked at 24 h post-dose, and then decreased with time. Next, liposome accumulation was compared in all four models after IV dosing (Fig. 5C). The 24 h post-dose time point showed peak accumulation across the models and was chosen for analysis. The highest percentage of liposome dose present in tumors was seen in the E35CR PDX model (Fig. 5C i), which possesses pericyte-covered vessels. Lower accumulation was observed in the other three models (Fig. 5C i), which lack pericyte-supported vessels, suggesting vessel support or maturity may enable improved nanomedicine delivery to tumors.

Tumor size can influence nanoparticle distribution, but was not a critical influence in the liposomal distribution patterns in the tumors examined: with both ITu and IV dosing, the overall trends remained similar when the percent of the injected dose was normalized for tumor volume (Fig. 5A ii and Fig. 5B ii).

### Detailed analysis of liposome localization in tumors

**Figure 6:**
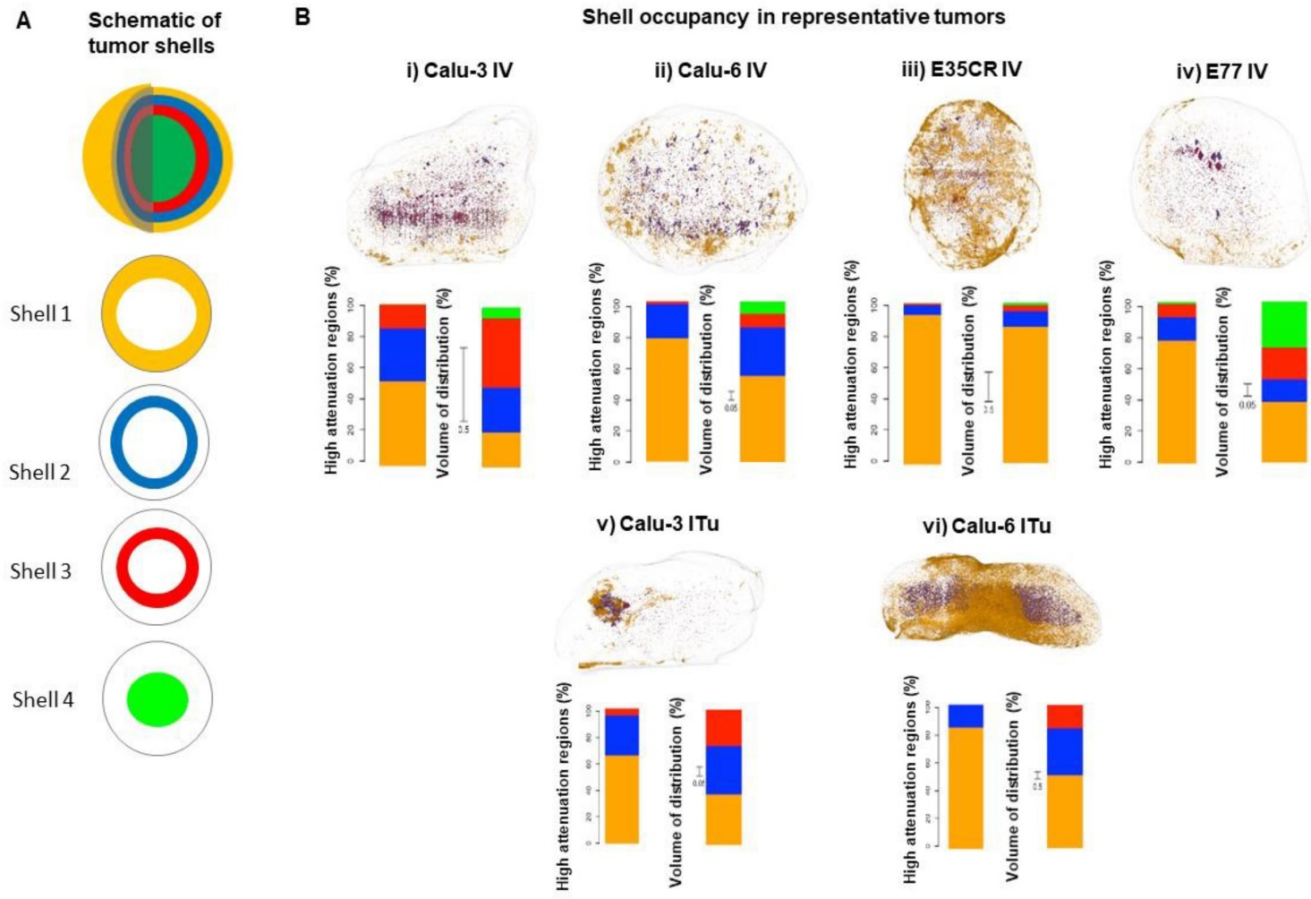
A) Schematic of tumor shells. For analysis, tumors were divided into three shells of 1 mm thickness beginning at the tumor periphery and moving towards the core, and a fourth shell encompassing everything greater than 3 mm from the periphery. B, images) Representative tumors showing liposome distribution in 3D, color-coded by shell across different tumor models at 24 h following IV (i-iv) or ITu dosing (v-vi). B, graphs) Quantification of liposome distribution across shells for shown tumor (left bar) and percentage of each shell volume occupied by liposomes (right bar, note: non-uniform scale). IV dosing (i-iv) and ITu dosing (v-vi).

As presented above, a similar total percentage of the injected dose accumulated in the Calu-3, Calu-6, and E77 tumors. Next, more detailed analysis was completed to determine whether the accumulated liposomes were similarly distributed in all three models. This was achieved by exploiting the unique ability of this imaging methodology to determine the liposome localization in 3D with semi-quantitative determination of accumulation at specific depths away from the tumor periphery. As depicted in the schematic (Fig. 6A), each 3D tumor image was segmented into shells, beginning at the tumor periphery (shell 1) and moving towards the tumor core (shell 4). The outer three shells were each 1 mm thick, while the fourth shell encompassed the remaining core of the tumor (beginning 3 mm from the tumor periphery). The liposome content in each shell was quantified to understand the depth of liposome localization away from the tumor periphery and the volume of tumor occupied by liposomes at different depths in each model (Fig. 6B). In representative tumors, these parameters were evaluated by determining the percentage of liposomes localized in each shell (Fig. 6B i-vi left bar) and the percentage of shell volume occupied by liposomes (Fig. 6B i-vi right bar). It must be noted that the total volume of the most peripheral shell (shell 1) is greater than the volume of the second shell, which is greater than the volume of the third shell, and this difference in shell volume is accounted for when calculating the percentage of each shell volume occupied by liposomes.

Following IV dosing (Fig. 6B i-iv), the dominant accumulation of liposomes was seen in the peripheral 1 mm of the tumor (shell 1), an observation which was consistent across all models. As noted before, E35CR tumors, with pericyte-supported vessels, showed the greatest overall accumulation, but a more uniform coverage of tumor volume across the shells was achieved in the Calu-3 tumor. The E77 tumor, presenting with a homogeneous vascular distribution but without pericyte-association, also showed relatively uniform occupation of shell volume, and had the greatest Shell 4 volume coverage of the four tumors evaluated. These findings demonstrate that the ex vivo imaging data can be used to semi-quantitatively define liposome localization at specific depths within the tumor. In the models studied, it appears that increased accumulation and liposome distribution beyond the tumor periphery may rely on the presence of supported vessels, potentially with improved functionality, at greater depths from the tumor edge.

When liposomes were administered via ITu dosing (Fig. 6B v-vi), the Calu-3 tumor showed more concentrated and deeper liposome localization, and the Calu-6 tumor showed greater liposome coverage of the peripheral two shells. As the distribution of liposomes administered via ITu dosing seems to be heavily influenced by the initial disposition immediately after dosing, it would be necessary to fully standardise injection depth, pressure, location, and speed to enable careful investigation of the role of the tumor microenvironment in liposome distribution, trafficking and retention following ITu administration.

### Evaluation of intra- and inter-model variability when analysing depth of liposome localization

**Figure 7:**
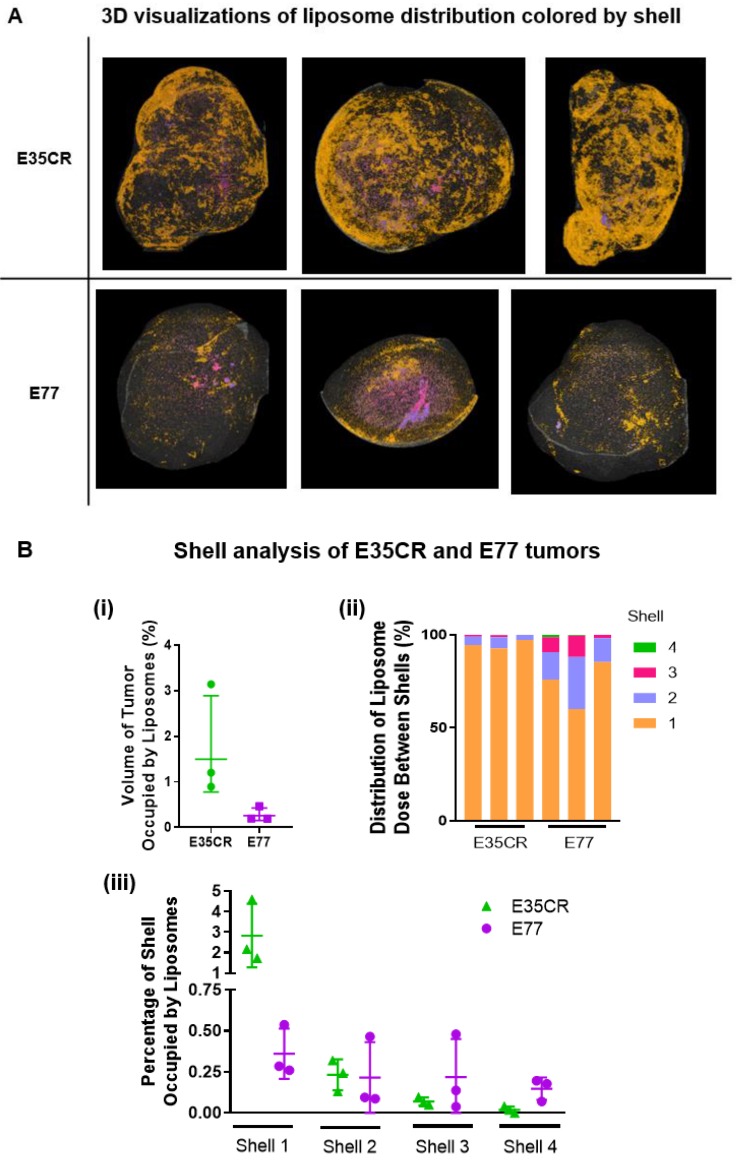
A) Liposome distribution in 3D, color-coded by shell, in E35CR and E77 tumors at 24 h post-IV dose. B) i) Graph showing total volume of tumor occupied by liposomes (%) in E35CR and E77 tumors (n = 3) at 24 h post-IV dose. Geomean +/- geoSD presented. ii) Distribution of tumor-accumulated liposomes between shells for individual E35CR and E77 tumors at 24 h post-IV dose. iii) Percentage of volume of each shell occupied by liposomes in individual E35CR and E77 tumors at 24 h post-IV dose.

The intra- and inter-model variability in the shell analysis was investigated across individual E35CR and E77 tumors following IV administration of liposomes. Consistent levels of accumulation and patterns of intra-tumoral distribution were observed between the three tumors of each model (Fig. 7A top, E35CR; bottom, E77). However, in contrast to the E77 tumors, the liposomes in E35CR tumors were shown to occupy a significantly larger total volume of the whole tumor (Fig. 7B i). The difference in vessels and associated liposome accumulation and distribution between these two prostate models suggest that vessel phenotype may be associated with liposome delivery. The pericyte-covered vessels in the E35CR tumors might be more mature than the pericyte-free vessels in the E77 tumors, and this could result in improved blood flow. Further to this point, liposomes occupied a greater percentage of shell 1 volume in the E35CR tumors than in the E77 tumors (Fig. 7B iii). A larger proportion of liposomes were localized at a greater depth (shells 2-4) in the E77 tumors than in the E35CR tumors (Fig. 7B ii), though the overall accumulation was lower. This translated to an increased percentage of the shell volume occupied by liposomes for shells 3 and 4 in E77 tumors than in E35CR tumors (Fig. 7B iii). This finding was unexpected based on the unsupported nature of the vessels in E77 tumors. However, this model presents with vessels throughout the tumor (Fig. 2). When considered with the highly focalized nature of the deeply distributed liposomes, it suggests a very small proportion of the vessels located in the tumor core delivered liposomes to core regions.

## Discussion

The assumption that the EPR effect is a universal feature of solid tumors and drives nanomedicine efficacy is beginning to be challenged. Nanomedicine accumulation, distribution and retention are critical determinants of therapeutic effect (in addition to sustaining peripheral pharmacokinetic exposure), and it is apparent that the tumor microenvironment affects treatment outcomes in preclinical models 26-31. Gaining insight into the influence of the tumor microenvironment on nanomedicine accumulation, distribution and retention, and thereby efficacy, will help guide more informed nanomedicine development programs 32.

High-resolution visualization of nanomedicine distribution in 3D is very challenging. Assessments of the loco-regional intratumoral distribution of nanomedicines in preclinical tumors has been limited by a dependence on classical methods, such as multi-section histology, or low-resolution live imaging techniques. To visualize nanomedicine distribution in 3D in whole tumors, we developed a semi-quantitative ex vivo µCT imaging method and applied it to four tumor models with diverse microenvironments. PEGylated liposomes (100 nm) loaded with the imaging agent iohexol were administered ITu and IV, and following careful excision of tumors and immediate imaging, liposome distribution was visualized in whole tumors with a spatial resolution of 17 µm.

Although small group sizes were used in the current study, the data were relatively consistent between individual tumors of each model and also at each time point within a model, enabling time course information to be derived in some experimental setups. To validate the µCT visualization data, we identified a well-established analysis technique that has been used to assess nanoparticle distribution in tumors: DESI-MSI 8. DESI-MSI was completed on sections of µCT-imaged tumors. In multiple tumors, iohexol-loaded liposomes were detected with a similar distribution pattern via both µCT imaging and DESI-MSI, showing strong concordance between the two techniques. Notably, MSI imaging requires twelve hours to scan one tumor slice at 20 µm resolution, with 20-40 slices per tumor, while an entire 3D tumor could be imaged in one hour using µCT. The combination of throughput and resolution achieved via the µCT method is critical for the assessment of 3D distribution in the context of the tumor microenvironment.

This semi-quantitative image analysis enables comparison of the relative level of liposome accumulation within and between tumors. However, it is important to recognise that the approach is impacted by the limits of detection of the contrast agent in areas of low liposome concentration, where liposomes may be present but at low abundance. Despite this caveat, the highest levels of liposome accumulation, which are likely to correspond to therapeutic levels of the nanomedicine, are reliably detected. Further developments of the technique could include the addition of algorithms capable of using the µCT absorption data to determine the absolute concentrations of the iohexol in tumors. Finally, free iohexol is rapidly cleared from circulation and did not reach detectable concentrations in tumors. Consequently, the analysis presented here focuses on the assessment of liposome accumulation and distribution trends rather than absolute quantities of iohexol in the tumor.

Following IV administration, this imaging method proved sufficiently sensitive to identify distinct patterns of liposome distribution at 24 h after IV administration in the four models tested. This time point was selected for distribution analysis as it corresponds to peak liposome accumulation in tumors 33-37. The method proved to be robust and discriminatory despite only testing small n numbers per model. Across all models, high peri-tumoral liposome accumulation was seen within the tumor capsule after 24 h, but delivery of liposomes beyond the tumor periphery varied with model. E35CR tumors showed the highest levels of liposome accumulation and were the only tumors with pericyte-associated vessels. In contrast, the E77 model is well-vascularized but possesses no vessel support (i.e., neither pericyte association nor stromal structure). In these tumors, low levels of liposome accumulation were observed despite the high vessel density, suggesting that vascular density alone does not suffice to enable efficient liposome delivery. Interestingly, small highly concentrated regions of liposomes were detected in the core of E77 tumors. This finding was unique to the E77 model and may indicate anti-cancer nanomedicines could achieve therapeutic drug levels, and cell death, in the tumor core in this tumor phenotype.

Very low accumulation was observed in the Calu-6 model. This may have been predicted as the model possesses a low vascular and stromal density, and high cellular density. Moreover, the vasculature in the Calu-6 model is limited to the tumor periphery, with the exception of highly compressed vessel structures in the core, which may have been unable to deliver liposomes in regions of high interstitial pressure. Based on the Calu-3 tumor microenvironment, the poor liposome accumulation observed following IV dosing was unexpected. Calu-3 tumors have homogeneously distributed vessels that are situated amongst dense stromal tracks. This complex stromal architecture might have been predicted to provide sufficient support for the vessels and prevent vascular compression, together enabling efficient liposome delivery. This, however, was not the case in these studies, perhaps suggesting that both vascular and stromal content vary between individual Calu-3 tumors and only an optimal balance will achieve efficient delivery of a carrier system.

Previously, Stapleton *et al*. 20 and Ekdawi *et al*. 11 used *in vivo* imaging techniques to assess the intratumoral distribution of liposomes in the ME180 cervix carcinoma model. Stapleton *et al*. showed that when the model was implanted orthotopically or intramuscularly, perfusion was an important parameter impacting the accumulation of liposomes in this tumor model. One limitation of this study was that the use of *in vivo* µCT imaging produced lower resolution visualizations that did not reveal the detailed heterogeneity in liposome distribution within the whole tumor. To address this, Ekdawi *et al*. also incorporated fluorescent microscopy on 2D tumor sections to assess liposome distribution in greater detail. This approach enabled further conclusions to be drawn supporting the importance of tumor vascular distribution in determining liposome distribution in the ME180 model. However, these are still lower resolution distribution data than are necessary to capture the heterogeneity in micro-distribution in 3D in the whole tumor.

To generate new insights into liposome distribution in whole tumors, we focused on visualizing liposome heterogeneity in the tumor in 3D and correlating this with detailed morphological assessments, including vascular and stromal density and tissue architecture. By imaging tumors ex vivo, we were able to achieve a significantly enhanced 3D resolution of 17 µm for liposome distribution in whole tumors. This advancement in resolution is obvious when viewing a rotating reconstruction of a 3D tumor following ITu dosing (video presented as Supplementary Figure S3). Moreover, we were able to improve the throughput of the technique to assess four preclinical models with significant morphological diversity. By careful preservation of the samples we directly compared the images acquired via 3D µCT with more classical analysis of multiple histological biomarkers. To enable comparison with the data from other published papers, we also included a 'shell' analysis. Our findings support the importance of vasculature, but also reveal that vessel support is a critical feature for liposome delivery, and that this only becomes apparent with the analysis of a panel of tumor models. Finally, the high-resolution 3D reconstructions show the extent of heterogeneity in liposome distribution within the whole tumor, which is challenging to represent with other techniques. Here we have imaged the distribution of iohexol-loaded liposomes, but the same technique could be applied more broadly to other drug delivery systems carrying an imaging agent.

Our findings support previous reports showing the importance of vascular flow in nanomedicine accumulation 13, 20 and the link between vessel distribution and the spatial distribution of liposomes 11. This study provides data that join detailed distribution in 3D to 2D histological feature analysis, and suggests that for any drug delivery system it is essential to investigate distribution at this level of resolution across multiple models. When drawing conclusions about accumulation, distribution or retention, it is necessary to assess diverse tumor microenvironment phenotypes. A limitation of the current study is that it does not assess classical tumor physiology parameters, such as perfusion or vessel permeability 38, and instead focused on histological analysis. Moreover, it was beyond the scope of the present work to incorporate an assessment of interstitial pressure, which is challenging to measure clinically. Where applicable, future development of the workflow could generate new insight by incorporating the techniques necessary to measure these parameters. For example, visualizing nanocarrier distribution preclinically and coupling this with identification of functional and non-functional vessels in the whole tumor could generate impactful understanding of the relationship between vessel function and delivery system accumulation and distribution in 3D. Clinically this may be possible through DCE-MRI perfusion assessments, or preclinically through the administration of fluorescent lectins to label functional vessels for detection via fluorescent microscopy ex vivo. Tumor histology and physiology have repeatedly been shown to correlate, though tumor-type specificity cannot be ignored. The approach evaluated here linked nanomedicine distribution with histology, which can be assessed clinically through biopsies.

Liposomes were administered ITu to test the methodology. These experiments resulted in interesting observations related to spatial changes in delivery system localization over time, as well as liposome retention and clearance in tumors. Three models were dosed ITu with liposomes. The liposomes were highly concentrated and non-uniformly distributed in all models. Others have demonstrated that a high interstitial fluid pressure limits liposome distribution 18. In our case, within each tumor model, the pattern of liposome distribution remained constant over time up to 24 h. This was surprising as we expected further liposome distribution or clearance post-injection. The fact that this was not observed may suggest an elevated interstitial fluid pressure that prevents significant carrier movement in tumors, or the absence of lymphatic-mediated clearance by 24 h. By 5 days post-dose, few liposomes remained in tumors. This suggests a slow but persistent loss of liposomes over time from the initially high concentrations delivered to the tumors.

In future, high-resolution 3D liposome visualization via µCT imaging would be most powerful when coupled to complementary methodologies. High-resolution 2D imaging techniques like imaging mass cytometry (CyTOF) could be employed in concert, to discriminate between liposomes retained within tumor vessels and liposomes that have extravasated out of the vasculature and reside in the tumor interstitium. CyTOF analysis of regions of high liposome abundance could help to identify the co-located cell types (macrophages, fibroblasts, vascular endothelial cells) or features of the surrounding extracellular matrix with single cell resolution. Novel insight into these relationships will improve the potential for nanomedicine applicability and translation.

In conclusion, we demonstrate that ex vivo imaging of iohexol-containing liposomes can give new understanding of the 3D patterns of accumulation and distribution in whole tumors. The contrast agent iohexol was useful for method development but the approach could be modified for different CT-based contrast agents or other delivery systems. This technique can be used to rapidly and accurately assess nanomedicine distribution across tumor models in 3D. It provides novel whole tumor visualizations that complement standard techniques such as MSI, bioanalysis, or routine 2D histological or microscopy approaches. This methodology to achieve high-resolution 3D visualization could be broadly applied to build new insight into the influence of the tumor vasculature and microenvironment on nanomedicine accumulation, distribution and retention. A more clinically relevant and translatable understanding of these relationships can guide the design of new nanomedicines under development, and their clinical application in specific patient populations or lines of sight where they are most likely to achieve therapeutic benefit.

## Supplementary Material

Supplementary figures and tables.

## Abbreviations

EPR: Enhanced Permeability and Retention
µCT: micro computed tomography
3D: 3-dimensional
PDX: patient-derived xenograft
2D: 2-dimensional
MSI: mass spectrometry imaging
DCE-MRI: dynamic contrast-enhanced-magnetic resonance imaging
IV: intravenous
ITu: intratumoral
IHC: immunohistochemistry
αSMA: alpha smooth muscle actin
DESI-MSI: desorption electrospray ionisation-mass spectrometry imaging
NSCLC: non-small cell lung carcinoma
CyTOF: imaging mass cytometry
