# Supplementary material for: High-resolution 3D visualization of nanomedicine distribution in tumors
Source: Theranostics. 2020 Jan 1;10(2):880–97. doi: 10.7150/thno.37178 (PMC6929971; doi:10.7150/thno.37178)
Supplement: Supplementary file 1 — Supplementary figures and tables. [file thnov10p0880s1.pdf]

Supplementary Materials

High-resolution 3D visualization of nanomedicine distribution in tumours

Jennifer I. Moss *et al*.

Figure S1. Representative whole tumor sections showing IHC staining for CD31 (top) and αSMA (bottom).

Suppl. Fig. 1A: Representative CD31 (top) and αSMA (bottom) IHC staining on whole tumor section of Calu-6 tumor.

CD31

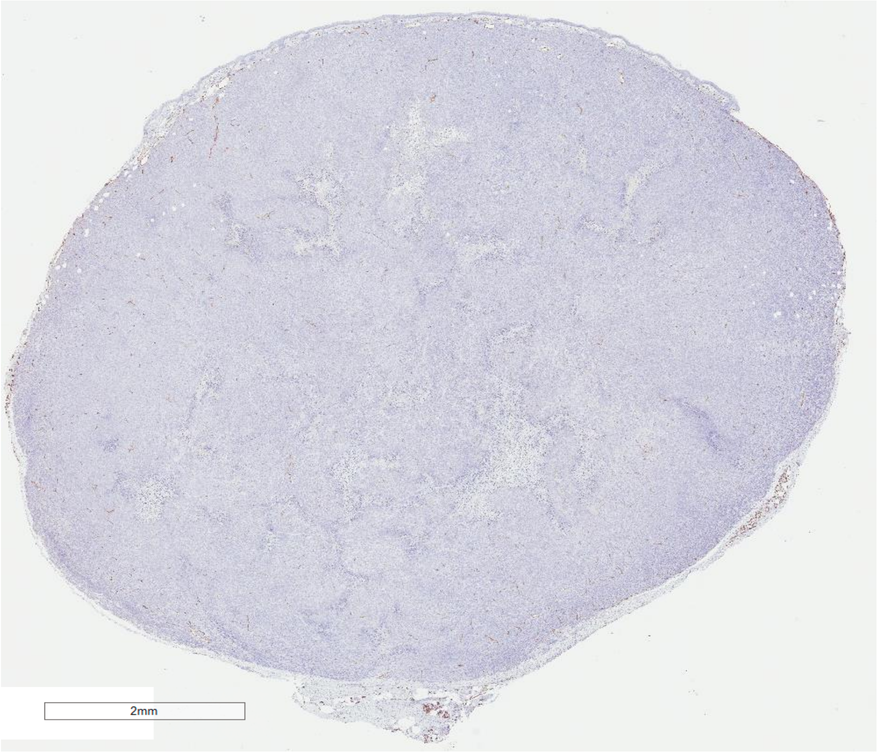

αSMA

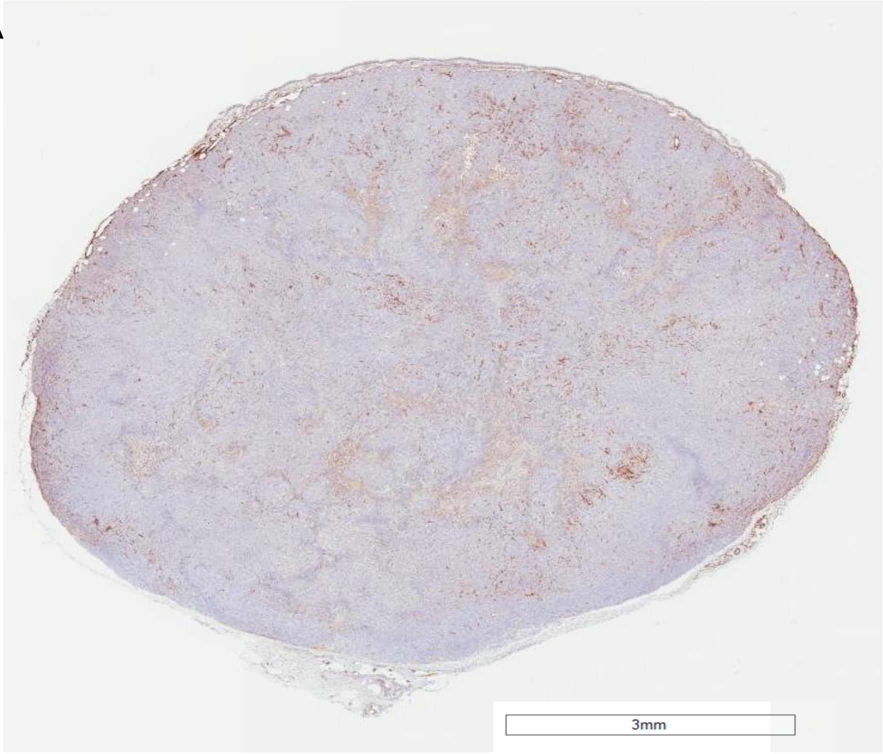

Suppl. Fig. 1B: Representative CD31 (top) and αSMA (bottom) IHC staining on whole tumor section of Calu-3 tumor.

CD31

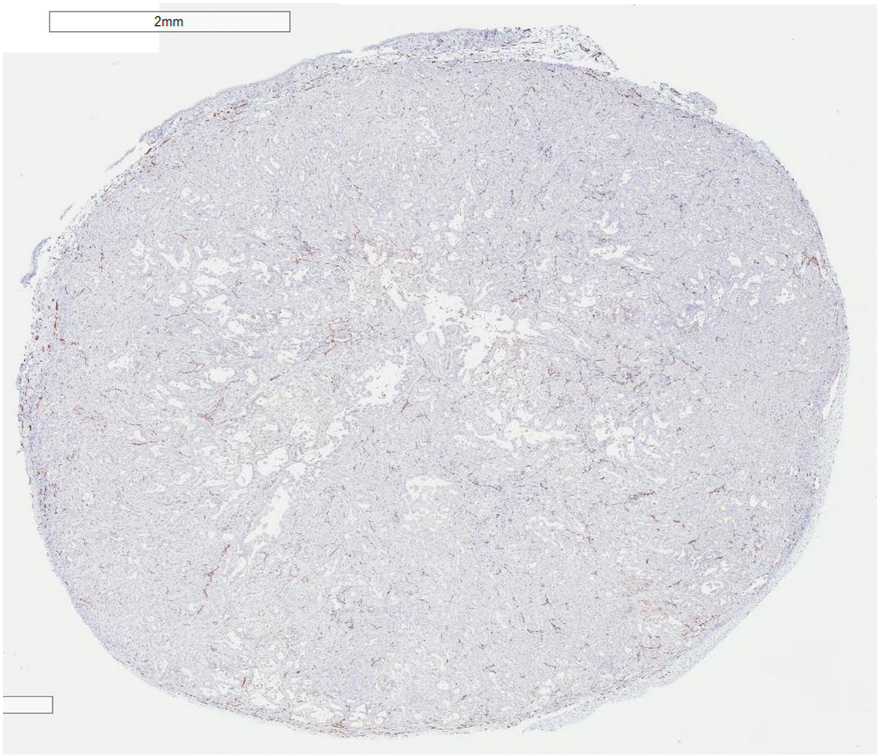

αSMA

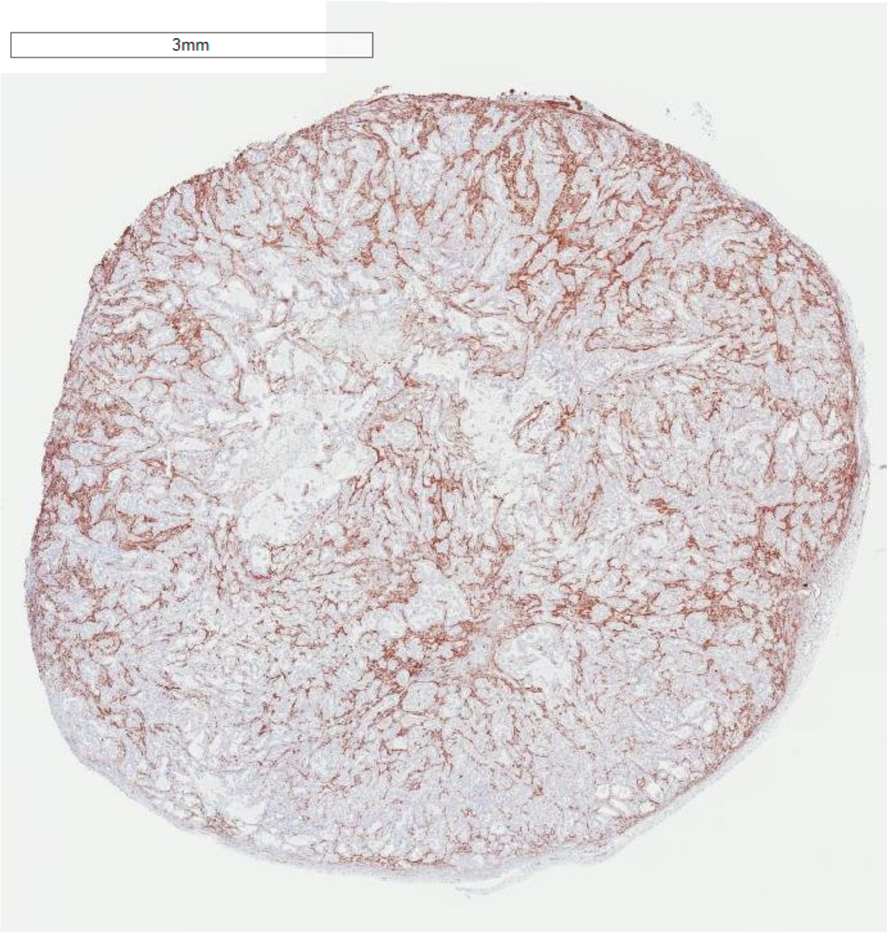

Suppl. Fig. 1C: Representative CD31 (top) and αSMA (bottom) IHC staining on whole tumor section of E35CR tumor.

CD31

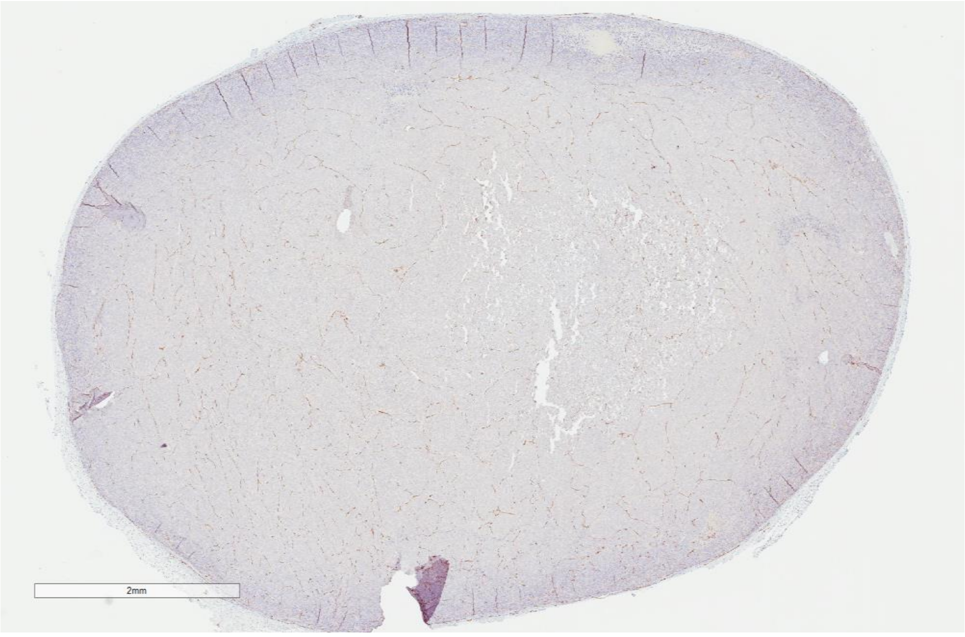

αSMA

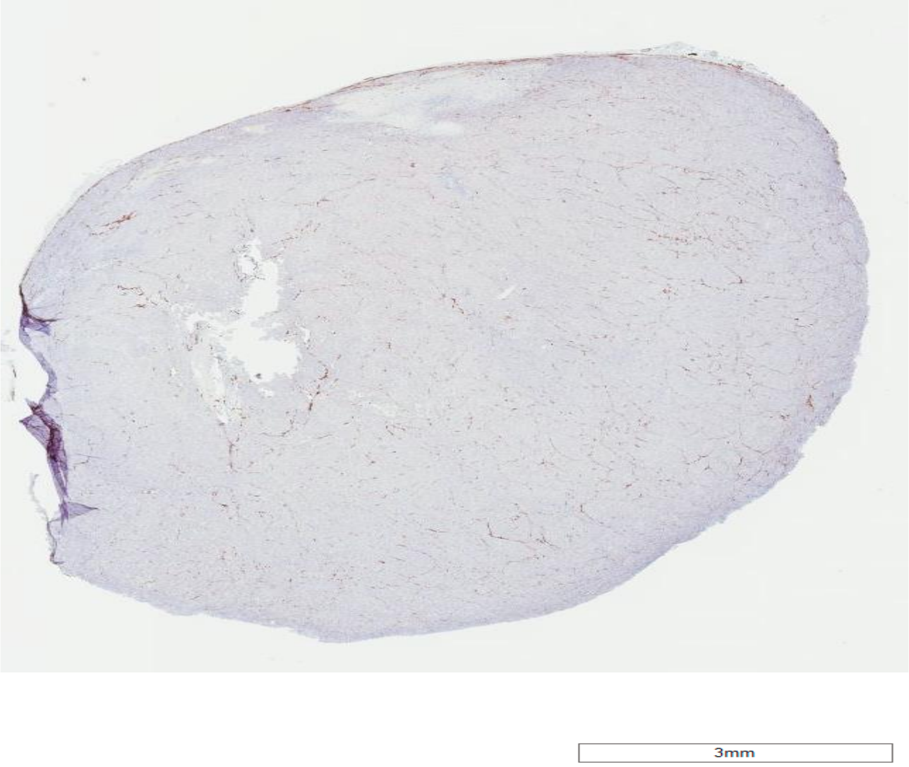

Suppl. Fig. 1D: Representative CD31 (top) and αSMA (bottom) IHC staining on whole tumor section of E77 tumor.

CD31

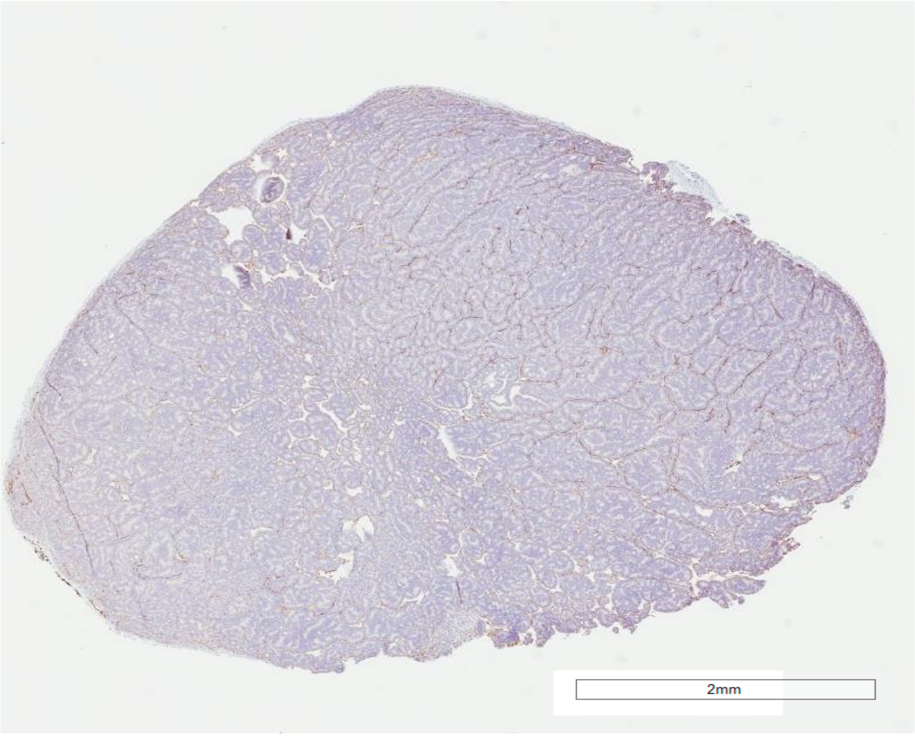

αSMA

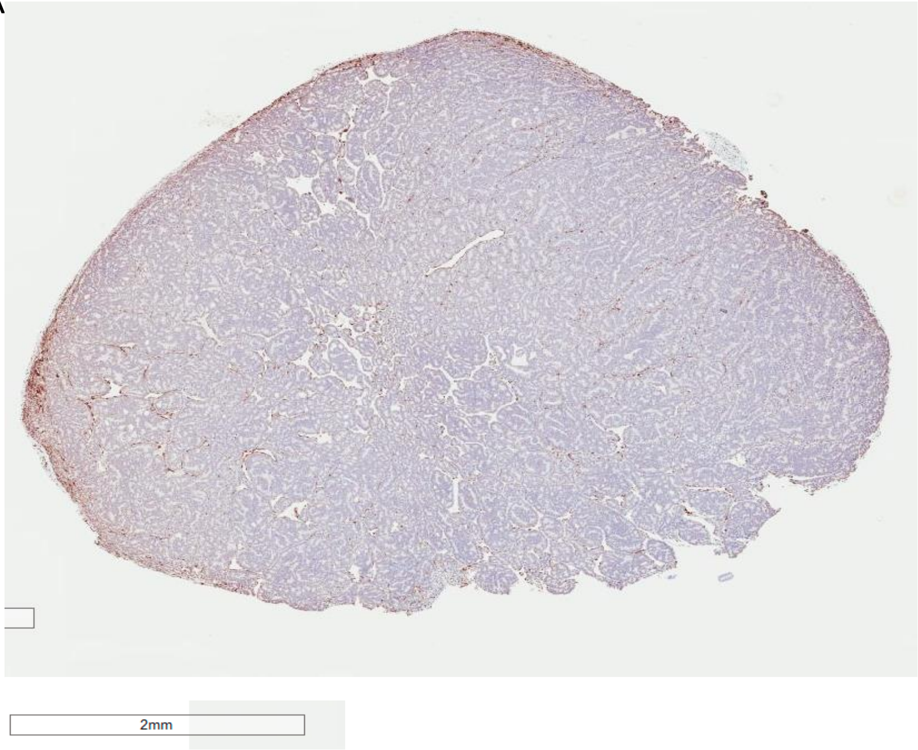

Figure S2. Lack of μCT enhancement in tumor at 30 minutes post-IV (A) and post-ITu (B) administration of free iohexol.

Suppl. Fig. 2A: Calu-6 tumor at 30 mins post-ITu dose of free iohexol.

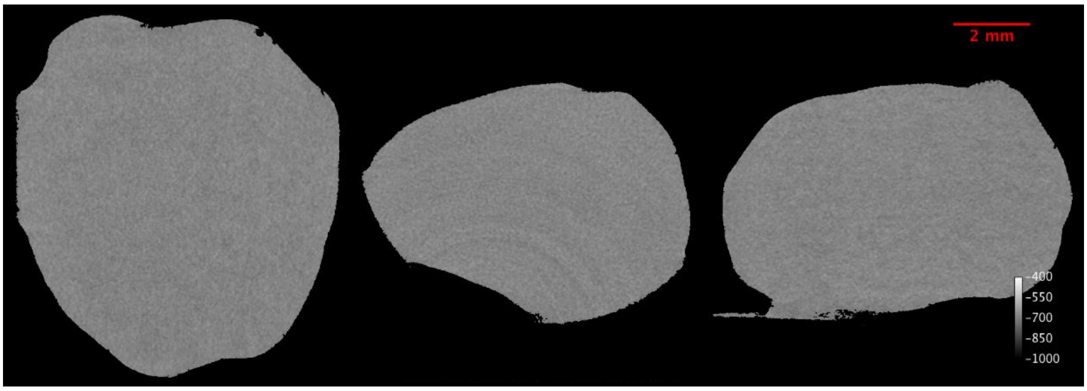

Suppl. Fig. 2B: Calu-6 tumor at 30 mins post-IV dose of free iohexol.

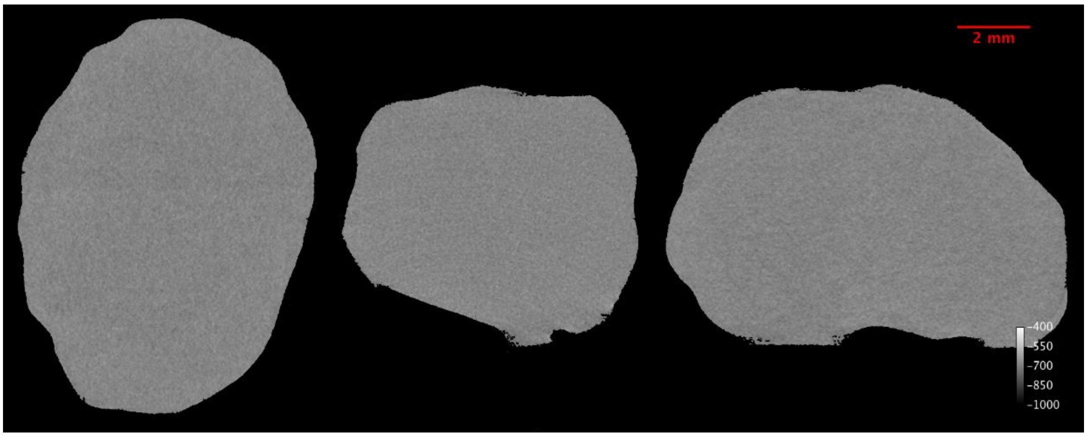

Figure S3. Rotating 3D visualization of liposomes in H358 tumor post-ITu dosing.

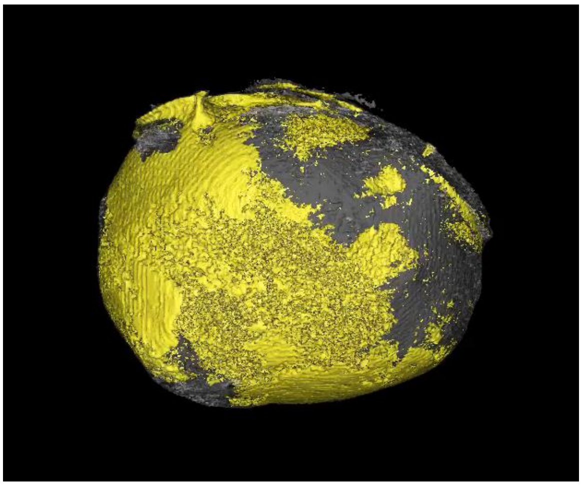

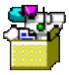

3D Visualisation
Rotating Tumour.mp4
